# X-ray crystallography of TRP channels

**DOI:** 10.1080/19336950.2018.1457898

**Published:** 2018-04-30

**Authors:** Appu K. Singh, Luke L. McGoldrick, Kei Saotome, Alexander I. Sobolevsky

**Affiliations:** a Department of Biochemistry and Molecular Biophysics, Columbia University, New York, NY; b Integrated Program in Cellular, Molecular and Biomedical Studies, Columbia University, New York, NY

## Abstract

Transient receptor potential (TRP) ion channels are molecular sensors of a large variety of stimuli including temperature, mechanical stress, voltage, small molecules including capsaicin and menthol, and lipids such as phosphatidylinositol 4,5-bisphosphate (PIP_2_). Since the same TRP channels may respond to different physical and chemical stimuli, they can serve as signal integrators. Many TRP channels are calcium permeable and contribute to Ca^2+^ homeostasis and signaling. Although the TRP channel family was discovered decades ago, only recently have the structures of many of these channels been solved, largely by cryo-electron microscopy (cryo-EM). Complimentary to cryo-EM, X-ray crystallography provides unique tools to unambiguously identify specific atoms and can be used to study ion binding in channel pores. In this review we describe crystallographic studies of the TRP channel TRPV6. The methodology used in these studies may serve as a template for future structural analyses of different types of TRP and other ion channels.

## Introduction

The first member of the transient receptor potential (TRP) channel family was identified as a gene in *Drosophila melanogaster* that, when mutated, caused visual impairment in fruit flies exposed to bright light [1, 2]. Since that first finding, TRP channels have been identified in different organisms, ranging from single cell organisms to vertebrates [3].

TRP channels can respond to a wide range of stimuli including temperature, membrane voltage, mechanical stress and a large array of chemicals [3, 4]. Correspondingly, TRP channels participate in a variety of physiological processes including noxious stimuli sensation, contraction and relaxation of smooth muscles, and homeostatic functions such as calcium reabsorption in the GI tract [3–5]. Based on sequence similarity, mammalian TRP channels are divided into six subfamilies: TRPC (canonical), TRPV (vanilloid), TRPM (melastatin), TRPP (polycystin), TRPML (mucolipin), and TRPA (ankyrin) [3]. All TRP channels are cation-selective but exhibit considerable variability in calcium permeability [6]. Two members of the vanilloid subfamily, TRPV5 and TRPV6, are permeable to calcium with high selectivity (P_Ca_/P_Na_ > 100), while others are weakly selective (P_Ca_/P_Na_ ≤ 12) or not calcium selective at all (TRPM4 and TRPM5) [7–9]. Importantly, mutations in TRP channels are implicated in a number of diseases including nociceptive and cardiovascular disorders, and different types of cancer [10–14].

Because of their diverse roles in physiology and pathophysiology, TRP channels represent important targets for structural analyses. Recently, advanced cryo-EM revealed a variety of structures from different TRP channel subfamilies [15–30]. Prior to the cryo-EM “resolution revolution” [31] attempts to use X-ray crystallography to solve TRP channel structures had limited success due to challenges associated with low expression of these proteins, their multi-domain oligomeric architecture and, as a result, their highly dynamic nature and biochemical instability. Nevertheless, the “divide and conquer” approach yielded several crystal structures of isolated TRP channel soluble domains [32–42], while protein engineering assisted solving two crystal structures of full-length TRP channels [30, 43–45]. Notably, X-ray crystallography provides unique tools to unambiguously identify ions for studies of ion channel selectivity and permeation, which are unavailable in cryo-EM. In this review, we discuss methods that we employed to solve the crystal structure of TRPV6. We focus on protein engineering and emphasize other strategies that were undertaken to improve the crystallization behavior of TRPV6.

### Protein expression and construct screening using FSEC

Generally, a prerequisite for protein structure determination by X-ray crystallography is the ability to obtain milligram quantities of a pure, structurally homogenous protein sample. For the efficient expression of mammalian membrane proteins, such as TRP channels, eukaryotic expression systems can be utilized. These expression systems can employ yeast, insect, or HEK-293 cells that in many cases provide proper posttranslational modifications, protein folding, protein trafficking, or specific host lipids, like cholesterol, which can stabilize a protein's transmembrane domain. To meet these requirements for efficient protein production, we developed protocols to express TRP channels in Sf9 insect cells and in suspension-adapted HEK 293S cells lacking N-acetyl-glucosaminyltransferase I (GnTI-) using the Bacmam system [45, 46].

**Figure 1.**
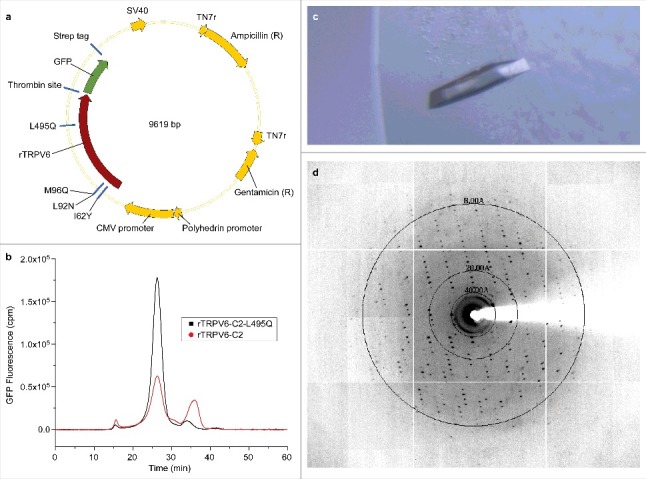
Expression, purification, and initial crystallization of rat TRPV6. (a) Map of the BacMam vector for rTRPV6 expression. The rTRPV6 construct has a C-terminal thrombin cleavage site followed by eGFP and a Strep affinity tag. The vector includes polyhedrin and CMV promoters, an ampicillin resistance selection marker, Tn7R transposition sites and an SV40 polyadenylation signal. (b) FSEC profiles for rTRPV6-C2 and rTRPV6-C2-L495Q constructs expressed in HEK 293 cells. (c–d) An image of an rTRPV6-C2-L495Q crystal in a hanging drop (c) and its diffraction pattern (d).

To identify suitable candidates for our crystallization experiments, we screened and biochemically characterized ∼20 different TRPV5 and TRPV6 orthologues. In these experiments, we used protein constructs that contained an N- or C-terminal enhanced green fluorescent protein (eGFP) tag, and either a poly-histidine or a WSHPQFEK streptavidin (Strep) affinity tag (Fig. 1a). Ultimately these tags were removed from the channel via a thrombin cleavage site introduced between the TRP channel and the eGFP tag. The presence of eGFP allowed us to use Fluorescence-detection Size Exclusion Chromatography (FSEC) [47], an assay that requires only nanogram quantities of a crudely extracted protein to evaluate its expression level, stability, monodispersity, oligomeric state and approximate molecular mass. FSEC is an outstanding tool to monitor protein behavior at different temperatures, in the presence of different detergents, lipids or ligands [47, 48]. This analysis is especially critical to assess the aforementioned qualities of engineered protein constructs with various modifications, including those with putative glycosylation sites knocked out, termini and flexible loops deleted, or containing tag insertions or point mutations [47–49]. When compared to other TRPV5 and TRPV6 channel orthologues, rat TRPV6 (rTRPV6) had the best FSEC profile in that it yielded a single, sharp, and monodisperse peak that eluted at the time expected for a tetramer solubilized in *n*-dodecyl-β-D-maltopyranoside (C_12_M) detergent (Fig. 1b). We interpreted this rTRPV6 FSEC profile as representing TRPV6 tetramers with uniform hydrodynamic mobility, which lacked either significant aggregation or dissociation into individual subunits. In the following section, we describe the steps that we took to engineer our crystallizing construct TRPV6_cryst_.

Our attempts to crystallize wild type rat TRPV6 (rTRPV6-wt) produced only low-quality crystals that did not diffract well. As a result, we made numerous modifications to rTRPV6-wt and ultimately engineered the TRPV6_cryst_ construct that produced crystals diffracting to a resolution of 3.25 Å [44].

### Crystallization trials of rTRPV6

Obtaining high quality-diffraction crystals of a membrane protein may require extensive construct modification. To improve crystallization of rTRPV6, we screened six N-terminal and seven C-terminal truncations and identified a construct, rTRPV6-C2, with 59 residues deleted from the C-terminus of rTRPV6-wt that was particularly biochemically stable. Additionally, a spontaneous mutation, L495Q, increased the expression level of rTRPV6-C2 approximately three-fold (Fig. 1b). Unless stated otherwise, this mutation was retained in all of our crystallization constructs. Protein purifications, primarily from Sf9 insect cells, comprised a membrane isolation step, membrane protein solubilization with C_12_M detergent, affinity chromatography with TALON or Strep resin, cleavage of the protein with thrombin to remove GFP and an octa-histidine or Strep tag, and a final gel filtration step with a Superose 6 column. The rTRPV6-C2 protein solubilized in C_12_M was subjected to high-throughput crystallization screening using a Mosquito robot. rTRPV6-C2 crystallized in various conditions containing low molecular weight polyethylene glycol (PEG) molecules (PEG 300, PEG 350MME, PEG 400, PEG 550MME) as precipitants. Ultimately, we found that PEG350 MME yielded the best, highly reproducible and large (> 200 μM-long) crystals (Fig. 1c) that grew in a hanging drop configuration and were shipped to synchrotron facilities for diffraction analysis. These crystals diffracted to a resolution of 6.2 Å (Fig. 1d) and subsequent indexing of the diffraction data showed that the crystals belonged to the C222_1_ space group. Various post-crystallization treatments [50] were also attempted on these crystals, including dehydration, soaking with different metal complexes, and covalent crosslinking by glutaraldehyde treatment. Unfortunately, none of these treatments enhanced the crystal diffraction quality. The resolution of 6.2 Å was insufficient for a detailed structural analysis of rTRPV6 and thus we continued to search for changes in the protein construct (terminal truncations, loop deletions, amino-acid substitutions, insertion of fusion partners, chimeras, different TRPV6 orthologs), alterations to our purification protocol (usage of alternative detergents, supplementary lipids or chemical additives, changes in buffer composition), crystallization conditions (detergent and additive screens, different crystallization techniques including under oil and lipidic cubic phase) or binding partners to aid in crystallization (e.g., anti-rTRPV6 antibodies). Some of these trials are discussed in the following sections.

#### Deletion of the extracellular S1-S2 loop

**Figure 2.**
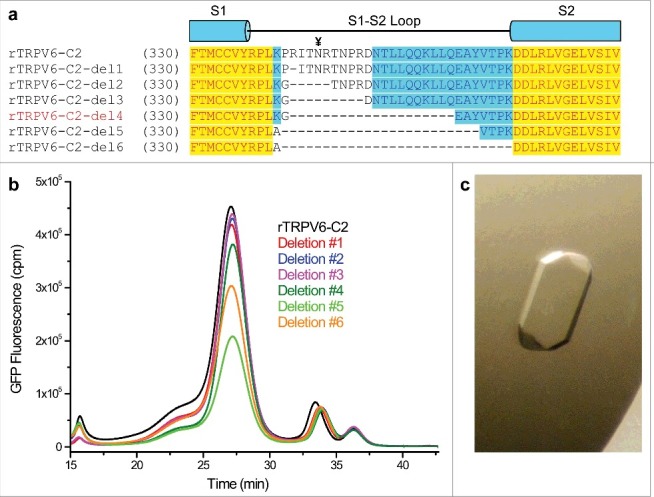
S1-S2 loop deletions. (a) Sequence alignment of the rTRPV6 constructs containing different size S1-S2 loop deletions. The glycosylation site is marked with the ¥ symbol. (b) FSEC profiles for the deletion constructs. (c) An example of an rTRPV6-C2-del4 crystal grown in a hanging-drop optimization tray.

When compared to TRPV1-4, both TRPV5 and TRPV6 have a long S1-S2 loop that contains a putative glycosylation site. Such a long and possibly flexible loop could potentially preclude the formation of well-ordered crystals. Thus, we made six constructs with a different number of residues removed from the S1-S2 loop (Fig. 2a). We accessed the expression and biochemical behavior of each construct using FSEC (Fig. 2b) and ultimately chose deletion #4 for further experimentation. This construct (henceforth referred to as RAD) had 21 residues in the S1-S2 loop (P354-L374) replaced with a single glycine, 13 residues truncated from the N-terminus, 59 residues truncated from the C-terminus and two cysteines mutated (C15S, C70A) to prevent non-specific disulfide bond formation. This construct had high expression and readily formed crystals in a number of conditions containing either high or low molecular weight PEGs (Fig. 2c). Similar to rTRPV6-C2, the best crystals of RAD belonged to the C222_1_ space group but unfortunately, never diffracted beyond 6.0 Å.

#### Crystal contact engineering

**Figure 3.**
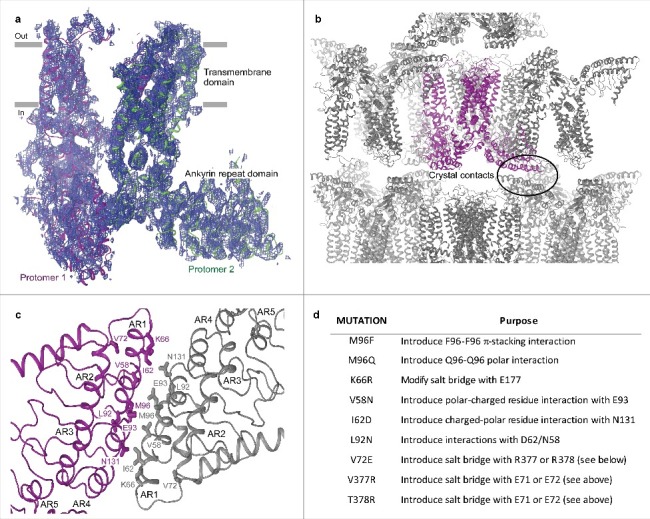
Low-resolution structure-driven engineering of crystal contacts. (a) Two protomers of rTRPV6-C2, representing the content of the asymmetric unit, viewed parallel to the membrane and colored purple and green. Blue mesh represents the electron density map at 1.0 σ. (b) Packing of rTRPV6-C2 into the C222_1_ space group crystal lattice. The protein content of the asymmetric unit is colored purple. The oval indicates the crystal contact formed between the ankyrin repeat domains. (c) Close-up view of the crystal contact with select residues shown in stick representation. (d) Table of mutations that were made to improve the crystal contacts.

Another approach to improve crystal diffraction is to strengthen the crystal contacts by engineering the surfaces of the protein that form these contacts. This approach relies on information about the crystal lattice, which can be obtained from low-resolution structural solutions. Unfortunately, we were unable to obtain a molecular replacement solution using TRPV1 as a search probe [21], in part because we only had low-resolution diffraction data, but also because of low (∼25%) homology between TRPV1 and TRPV6. We also attempted to solve the structure using the rat TRPV6 ankyrin repeat domain (ARD) crystal structure (PDB ID: 2RFA) [36] as a search probe for molecular replacement but were unsuccessful again, likely because the ARD represent only a small (∼33%) portion of the protein. To increase the protein mass of the search probe, we fused the rat ARD with the TRPV6 S1-S4 domain homology model generated based on the TRPV1 cryo-EM structure [21]. We tried different molecular replacement methods including those implemented in PHASER [51], MOLREP [52], and AMoRe [53], but those attempts were unsuccessful. Fortunately, we came across the MRAGE [51, 54] module in PHENIX [55] that finally resulted in a plausible structural solution that showed the C222_1_ space group packing of two TRPV6 protomers in an asymmetric unit (Fig. 3a), with the tetramer revealed by applying two-fold crystallographic symmetry. Analysis of the crystal packing identified the first three ankyrin repeats as being extensively involved in crystal contacts (Fig. 3b). Since the high-resolution crystal structure of the rat TRPV6 ARD [36] was used for molecular replacement, the molecular model of these crystal contacts was accurate at the level of individual amino acids (Fig. 3c).

**Figure 4.**
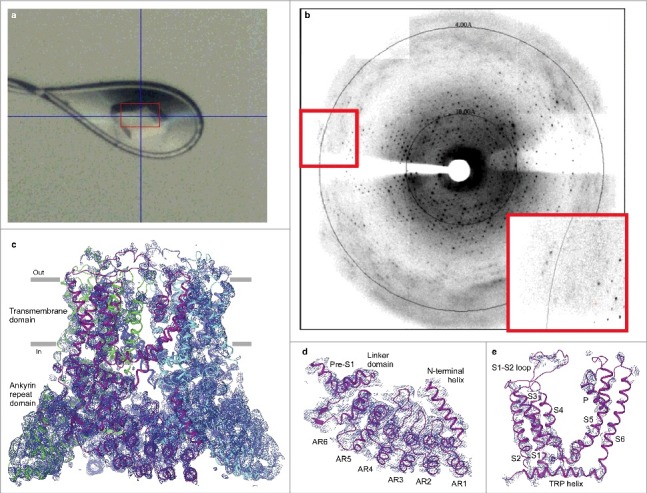
Crystallization of the RAD-NQ construct. (a) Example of an optimized RAD-NQ crystal flash frozen in a cryo-loop. The red box indicates boundaries of the X-ray beam. (b) 3.85 Å-resolution diffraction pattern for the RAD-NQ crystal. Inset is a magnified view of the diffraction pattern (red boxes). (c) Side view of the RAD-NQ tetramer that represents the content of the asymmetric unit. Each subunit is colored differently. Blue mesh represents the electron density map at 1.0 σ. (d–e) Close-up views of the electron density for the (d) ankyrin repeat and (e) transmembrane domains.

We sought to exploit this detailed knowledge of the crystal contacts to improve the diffraction pattern of our crystals. Specifically, we identified hydrophobic amino acids (Leu, Met, Ile, Ala and Val) residing at the crystal contact interfaces that were not involved in any interaction and mutated them to residues that could form hydrogen bonds (Fig. 3d). The majority of the new constructs did not yield crystals or produced similarly or worse diffracting crystals. After screening ∼40 constructs with single or combined mutations, and purifying them in different detergents and lipids, we were able to identify a pair of substitutions, L92N and M96Q, that produced a dramatic change in the crystallization behavior of rat TRPV6 purified in C_12_M in the presence of the soluble cholesterol analogue cholesteryl hemisuccinate (CHS). The new construct (RAD-NQ) crystalized in a variety of PEG-containing conditions and yielded beautiful 3-dimentional crystals (Fig. 4a) that diffracted anisotropically with reflections reaching beyond 4 Å (Fig. 4b).

Interestingly, the improved resolution coincided with the space group changing from C222_1_ for RAD to C2 for RAD-NQ. The structure, solved by molecular replacement using the same search probe as described above, revealed four TRPV6 protomers, or one tetramer, per asymmetric unit (Fig. 4c). The crystal lattice was formed by columns of the RAD-NQ tetramers interacting in a ‘head-to-head’ and ‘tail-to-tail’ manner, with the lateral contacts between the columns mediated by the ARDs. Inspection of the ‘tail-to-tail’ contacts identified potential interactions between the side chains of M96Q and Q59 from the opposing tetramers, which emphasized the importance of the M96Q mutation for this crystal contact. The importance of the L92N mutation was unclear, as it was apparently not involved in the crystal contacts. Multiple cycles of structural refinement using PHENIX [55] and REFMAC [56], and manual model building in COOT [57], revealed excellent density for the entire TRPV6 soluble region (Fig. 4d), including the ARD, ARD-S1 linker domain, N-terminal helix and C-terminal domain, and showed some structural features that were unseen in other TRPV channel structures [16, 19, 20]. However, our refinement efforts did not yield good density for the transmembrane domain, especially for the channel core, which includes the transmembrane helices S5 and S6 and the selectivity filter (Fig. 4e). Scattered density in the pore-forming domain was an obvious barrier to further analyzing the structure and function of TRPV6. Thus, we had to either improve the C2 crystal form or find alternative crystal forms in which clear densities could be seen for the transmembrane domain core.

#### Reversal of L495Q mutation

We reasoned that the scattered density in the transmembrane region could have resulted from the C2 symmetry that allowed close crystal packing with the ‘head-to-head’ and ‘tail-to-tail’ contacts. As we described previously, the L495Q mutation in S5 had occurred spontaneously during our construct engineering and fortuitously increased protein expression. This was the only mutation in the transmembrane domain region, in close proximity to the scattered density, which could potentially alter the channel folding. To test the effect of the L495Q mutation, we reversed it to the native leucine and grew crystals in similar conditions using the hanging drop configuration. The resulting crystals diffracted to 4.1 Å and belonged to the same C2 space group. The corresponding structure was almost identical to that of RAD-NQ and still exhibited no interpretable electron density for the transmembrane domain core.

#### Pore loop deletions

Upon inspection of the C2 crystal form packing, we hypothesized that the tight head-to-head contacts between the pore loops of symmetry mates might cause non-specific interactions and result in the poor electron density observed for the transmembrane domain core. Interestingly, sequence analysis of the pore loop region revealed the presence of a highly negatively charged amino acid stretch that could potentially destabilize the pore through electrostatic repulsion in the head-to-head crystal packing. Therefore, we targeted these negatively charged residues by making deletions in the RAD-NQ construct. In engineering these new constructs, we either shortened the S5-P loop by five residues (514-EDPDE-518, RAD-NQ-PD1 construct) or the P-S6 loop by four residues (546-YDVD-550, RAD-NQ-PD2 construct). Both of these constructs formed stable tetramers and were purified and crystallized in similar conditions as RAD-NQ. The corresponding structures showed the same uninterpretable electron densities for the transmembrane domain core, indicating that either the pore loops were not short enough or that packing in the C2 space group was causing the disorder, irrespective of the pore loops. Of note is that the channel core was preserved in the low-resolution rTRPV6-C2 structure obtained in the C222_1_ space group, which suggested that the rTRPV6 channel was not inherently disordered. Therefore, it appeared that the crystal packing caused distortion of the transmembrane domain region and resulted in the low quality electron density maps. Another possibility is that the S1-S2 loop deletion in the RAD-NQ construct allosterically perturbed the central pore structure.

#### Transplantations of pore loops from other ion channels

**Figure 5.**
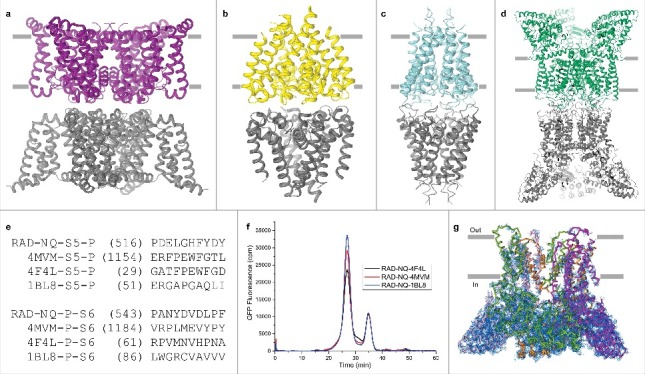
Pore loop transplantation from different ion channels. (a–d) Crystal contacts mediated by pore loops for (a) engineered voltage-gated calcium channel (PDB ID: 4MVM), (b) voltage gated sodium channel (PDB ID: 4F4L), (c) potassium channel KcsA (PDB ID: 1BL8) and (d) RAD-NQ. All structures were solved in the C2 space group. (e) Replaced sequences of the RAD-NQ pore loops aligned with the corresponding regions of the aforementioned channels. (f) FSEC profiles for different RAD-NQ pore loop chimera constructs. (g) Side view of the RAD-NQ-4MVM pore loop chimera tetramer that represents the content of the asymmetric unit. Each subunit is colored differently. Blue mesh represents the electron density map at 1.0 σ.

Subsequently, we searched the PDB for tetrameric channel structures that were solved in the C2 space group and that had well-resolved, pore loop-mediated, head-to-head crystal contacts. Those included calcium (PDB ID: 4MVM) [58], sodium (PDB ID: 4F4L) [59] and potassium (PDB ID: 1BL8) [60] channel structures (Fig. 5a–d). In an attempt to create improved crystal contacts, we transplanted the pore loops from these structures into the RAD-NQ construct and the resulting three chimeras, RAD-NQ-4F4L, RAD-NQ-1BL8 and RAD-NQ-4MVM (Fig. 5e), produced biochemically stable tetramers, similar to RAD-NQ (Fig. 5f). Unfortunately, crystals for each of these constructs diffracted to 4.0 – 4.3 Å resolution and resulted in the same electron density pattern as was observed for the RAD-NQ crystals (Fig. 5g).

#### Ankyrin repeat 5-fusion constructs

To better resolve the transmembrane domain core, we searched for alternate crystal forms by trying different TRPV6 constructs with point mutations and loop deletions. Unfortunately, most of these constructs exhibited similar or worse crystallization behavior, and so we decided to introduce more drastic changes. One strategy to increase the chances of finding new crystal forms is to insert small protein domains that can act as new scaffolds for crystal formation. This approach has been successfully utilized to crystallize many GPCRs [61–63].

**Figure 6.**
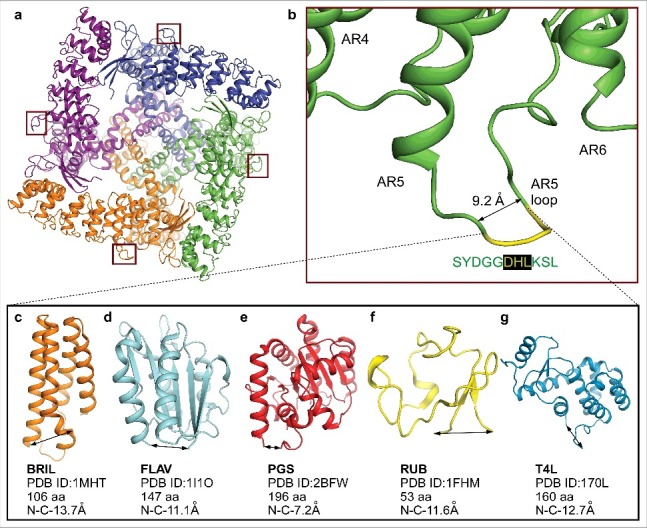
Ankyrin repeat fusion constructs. (a) Bottom view of the RAD-NQ tetramer with the ankyrin repeat 5 loop boxed and each monomer shown in a different color. (b) Magnified view of the ankyrin repeat 5 loop. The amino acid sequence (yellow) was replaced in each of the fusion constructs with a fusion partner. (c–g) Fusion partners inserted in the ankyrin repeat 5 loop: (c) BRIL (PDB ID: 1MHT), (d) FLAV (PDB ID: 1I1O), (e) PGS (PDB ID: 2BFW), (f) RUB (PDB ID: 1FHM) and (g) T4L (PDB ID: 170L). The double-headed arrows represent the N- to C- termini distances.

**Figure 7.**
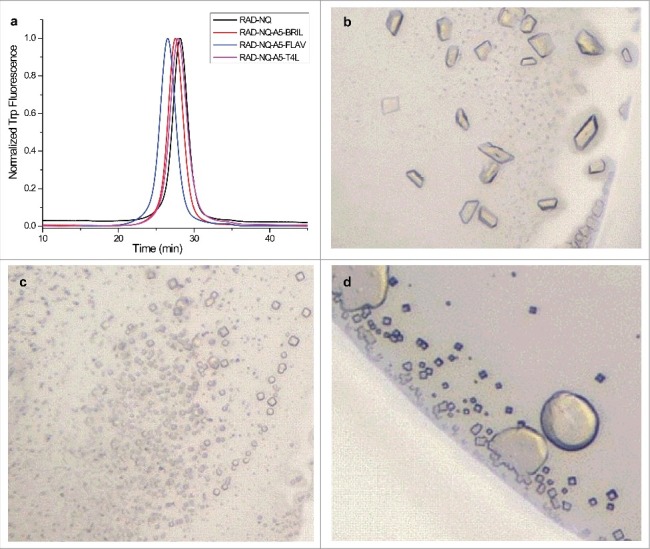
Purification and crystallization of the ankyrin repeat fusion constructs. (a) FSEC profiles for the purified ankyrin repeat fusion constructs. (b–d) Optimized crystals of the (b) T4L, (c) BRIL and (d) FLAV fusion constructs.

We inserted various small soluble proteins, including thermostabilized cytochrome B562RIL (BRIL, PDB ID: 1M6T), flavodoxin (FLAV, PDB ID: 1I1O), glycogen synthase (PGS, PDB ID: 2B45), rubredoxin (RUB, PDB ID: 1FHM) and the C-terminal portion of T4 lysozyme (T4L, PDB ID: 2O7A), into the ankyrin repeat 5 loop. This is the longest and the least conserved loop in the ARD, which protrudes from the periphery of the square-shaped soluble domain of rTRPV6 (Fig. 6) [63]. Despite differences in the folds of the inserted proteins, and their respective N-to-C-terminal distances, each of the fusion constructs displayed high expression and yielded an excellent FSEC profile (Fig. 7a). As expected, the fusion construct FSEC peaks were leftward shifted when compared to that of the “parent” RAD-NQ construct. We succeeded in growing crystals of RAD-NQ-A5-T4L, RAD-NQ-A5-FLAV, and RAD-NQ-A5-BRIL (Fig. 7b–d); however, these crystals grew in large clusters and never diffracted well.

#### Crystallization of rTRPV6-C2 and the route to a new crystal form

**Figure 8.**
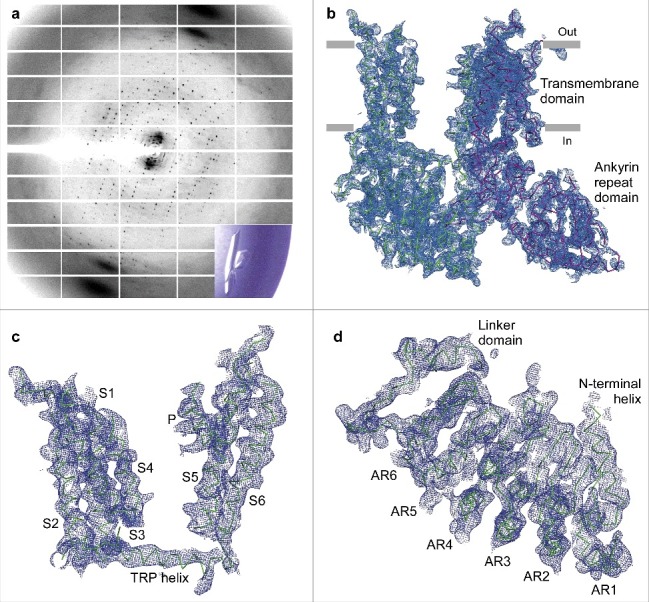
Crystallization of the rTRPV6-C2-NQ construct. (a) ∼4 Å-resolution diffraction pattern for the rTRPV6-C2-NQ crystal shown in the lower right corner inset. (b) Two protomers of rTRPV6-C2-NQ representing the content of the asymmetric unit, viewed parallel to the membrane and colored green and purple. Blue mesh represents the electron density map at 1.0 σ. (c-d) Close-up views of the electron density for the (c) transmembrane and (d) ankyrin repeat domains.

As previously described, we identified two amino acid substitutions, L92N and M96Q, which resulted in a remarkable improvement of RAD-NQ crystallization and diffraction resolution. Because we continued to struggle to improve the density of the transmembrane domain core, we decided to take a step back and introduce these mutations into the initial rTRPV6-C2 construct, which yielded crystals in the C222_1_ space group that diffracted to 6 Å resolution. Importantly, while the original rTRPV6-C2 crystals diffracted to a low resolution, the resulting electron density was homogenous throughout the protein (Fig. 3a). The introduction of L92N and M96Q into the rTRPV6-C2 background (rTRPV6C2-NQ) resulted in higher quality crystals that diffracted to 4.0 Å (Fig. 8a). However, the rTRPV6C2-NQ structural solutions were difficult to refine, as was evidenced by an inability to obtain lower than 38% values for the crystallographic parameter R_free_. Also, despite the continuous density throughout the transmembrane domain core (Fig. 8b–c), the density for portions of the ankyrin repeat domain was relativley weak (Fig. 8d). Athough we tried altering the purification protocol and crystallization conditions, and performing additive screening, we were unable to improve the diffraction resolution and map quality. Because we had previously learned that manipulating the crystal contacts by mutagenesis could result in drastically different crystallization behavior, we attempted to use this approach again.

**Figure 9.**
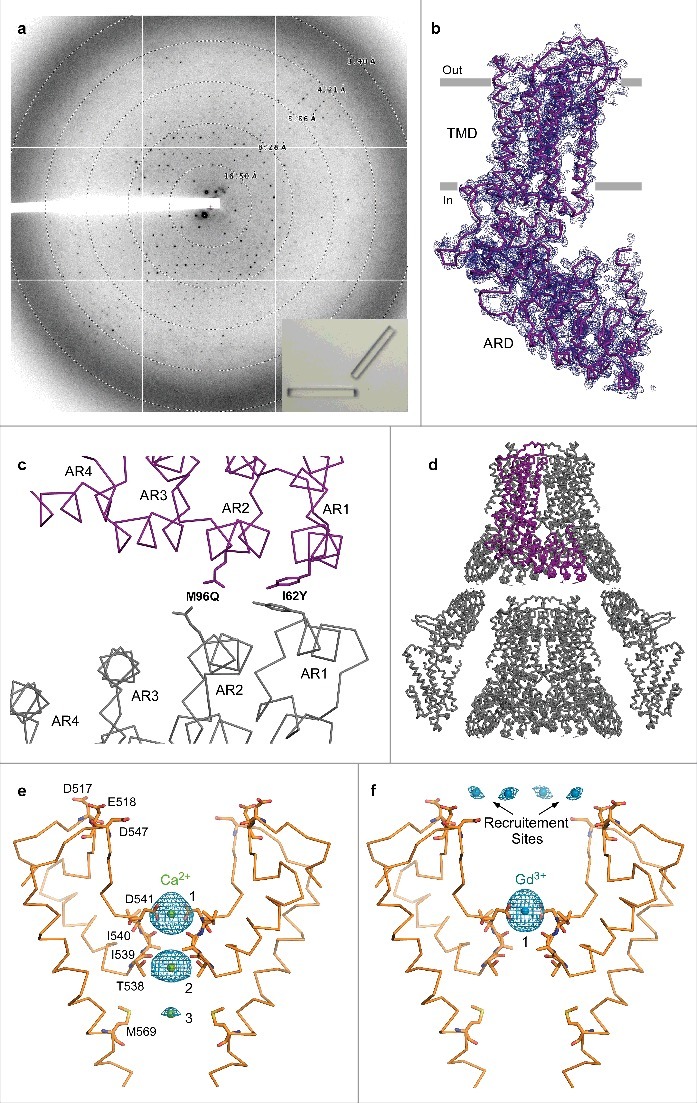
Crystallographic analysis of TRPV6_cryst_. (a) ∼3.25 Å-resolution diffraction pattern for the TRPV6_cryst_ crystal shown in the lower right corner inset. (b) TRPV6_cryst_ monomer representing the content of the asymmetric unit, viewed parallel to the membrane. Blue mesh represents the electron density map at 1.0 σ. (c) Close up view of the TRPV6_cryst_ crystal contact with the side chains of I62Y and M96Q shown in stick representation. (d) Orthogonal view of the TRPV6_cryst_ P42_1_2 space group crystal lattice. The protein content of the asymmetric unit is colored purple. (e-f) Ribbon models of the TRPV6_cryst_ pore with bound Ca^2+^ (e, green spheres) or Gd^3+^ (f, blue spheres). Only two of four subunits are shown with the front and back subunits omitted for clarity. Residues important for cation binding are shown as sticks. Blue mesh represents the anomalous difference Fourier maps generated from diffraction data collected at 1.75 Å wavelength for Ca^2+^ (e, 2.3 σ) and 1.56 Å wavelength for Gd^3+^ (f, 8.0 σ).

Three pairs of residues between symmetry mates resided in close proximity to one another at the C222_1_ crystal contacts of rTRPV6C2-NQ (Fig. 3b–c): (1) E93 and E93, (2) Q96 and Q96, and (3) I62 and N131. Assuming that the E93-E93 proximity could only result in unfavorable electrostatic repulsion, we mutated E93 to Q or N. In order to strengthen the interaction with N131, we mutated I62 to one of three polar or charged residues, N, D, or Y. The I62 mutations had strong effects on crystallization. Crystals of I62Y, grown in the low molecular weight PEG 350MME, resembled sharply edged bars, while I62N and I62D crystals look like flimsy needles. Optimization of the rTRPV6-C2-NQ-I62Y crystal growth conditions (100 mM NaCl, 100 mM Tris pH-8.0, 20–24% PEG 350MME) yielded large thin plate crystals that grew in hanging drops perpendicularly to the cover slide surface. The optimized crystals diffracted significantly better than our previous crystal forms, with diffraction spots observed beyond 3.5 Å resolution. However, these crystals were very thin and many of them became damaged during cryo-protection. In order to make these crystals more 3-dimentional, we screened nearly 400 additives and found one, sodium formate, which remarkably improved crystal thickness. Correspondingly, these crystals diffracted better and the diffraction spots were observed beyond 3.2 Å resolution and the data reduction resulted in 3.25 Å Bragg spacing (Fig. 9a).

The rTRPV6C2-NQ-I62Y construct (henceforth referred to as TRPV6_cryst_) crystallized in the P42_1_2 space group with one protomer per asymmetric unit (Fig. 9b). This was different from the previous rTRPV6 crystals forms, C222_1_ and C2, which had two or four protomers per asymmetric unit, respectively. Similarly, however, the crystal packing was mediated by the helical portions of the first two ankyrin repeats between the neighboring symmetry mates. Notably, the side chains of Y62 from each symmetry mate interacted with each other, clearly demonstrating how the I62Y mutation helped to form better crystals (Fig. 9c–d).

### Identification of ion binding sites along the TRPV6 channel pore

Ion channels execute their primary functions by allowing ions to permeate cell membranes. X-ray crystallography, as opposed to cryo-EM, is a structural biology technique that can be used to detect anomalous signals originating from ions to identify their binding sites inside the channel pore. To gain structural insight into the mechanism of TRPV6 channel ion permeation, we collected anomalous diffraction data from crystals grown in the presence of different ions at the wavelengths that correspond to the maximum anomalous signal for each of these ions. By taking advantage of the anomalous signals, we have visualized calcium, barium and gadolinium binding in the pore of TRPV6_cryst_. We discovered three binding sites in the center of the TRPV6_cryst_ pore for the permeant ions calcium and barium (Fig. 9e). For the stronger anomalous scatterers, barium and gadolinium, we also revealed four recruitment sites in the ion channel extracellular vestibule that are important for ion permeation (Fig. 9f). For gadolinium, which acts as a blocker of TRPV6 channels, we observed a strong anomalous signal at the main binding site in the pore formed by the D541 side chains (Fig. 9f), but no signals at the other two pore sites identified for the permeant ions. Based on the strength and location of the detected anomalous signals, we proposed a model of permeation and block of TRPV6 channels [43, 44] that will serve as a basis for future structural and functional studies.

### Domain swapping in TRPV6 channel

**Figure 10.**
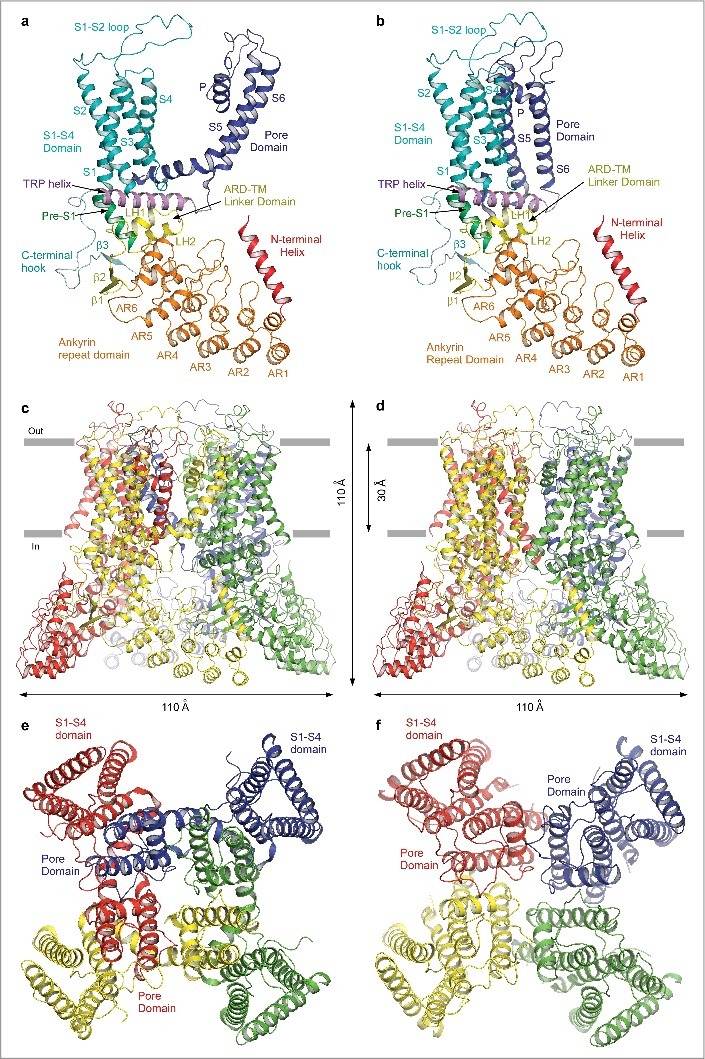
Swapped and non-swapped transmembrane domain arrangements in TRPV6. (a–b) Swapped TRPV6* (a) and non-swapped TRPV6_cryst_ (b) monomers are colored according to their domains. (c–f) Swapped TRPV6* (c,e) and non-swapped TRPV6_cryst_ (d,f) tetramers viewed parallel to the membrane (c–d) or extracellularly (e–f), with subunits shown in different colors. The S1-S4 domains are adjacent to the pore domains from the same subunits in TRPV6_cryst_, but are adjacent to the pore domains from neighboring subunits in TRPV6*.

While building the TRPV6_cryst_ structural model [44], we were surprised to discover that the resulting structure exhibited a non-swapped domain architecture, in which the S1-S4 and pore domains of a single protomer are located adjacent to each other (Fig. 10). Although the S4-S5 linker was disordered and not readily apparent in the electron density of TRPV6_cryst_, this non-canonical domain arrangement was supported by density representing the S6-TRP helix linker as well as cysteine crosslinking experiments. While the subsequent studies of HCN [64], CNG [65], Slo1 [66], Slo2.2 [67] and Eag1 [68] justified the legitimacy of the non-swapped transmembrane domain architecture in tetrameric ion channels, the other members of the TRP channel family exhibited only swapped architectures [16, 19, 20, 24, 38]. We were puzzled by this difference and decided to test whether L495Q, the only mutation in the TRPV6_cryst_ transmembrane domain, could oblate the domain swapping. We grew crystals of TRPV6_cryst_ with reinstated L495 (we named this construct TRPV6*) in the same crystallization conditions as TRPV6_cryst_ and solved the TRPV6* structure at 3.25 Å resolution [43]. To our great surprise, despite the overall shape of the TRPV6* molecule was indistinguishable from the shape of TRPV6_cryst_, it exhibited a domain-swapped fold reminiscent of other TRP channel structures [16, 19, 20, 24, 38], but different from the non-swapped fold of TRPV6_cryst_.

The domain-swapped arrangement of TRPV6*, in which the S1-S4 and pore domains of different protomers are adjacent to each other, was substantiated by robust density and clear connectivity between the S4 and S5, as well as the S6 and TRP helices. Apart from the S4-S5 and S6-TRP helix linkers, the domain-swapped TRPV6* and non-swapped TRPV6_cryst_ structures are almost identical. Superposition of these structures, excluding the linker regions (residues 470–585), results in the root mean square deviation, RMSD = 0.542 Å. Notably, the architecture of the TRPV6* and TRPV6_cryst_ pores, including the selectivity filter and pore-lining S6 helices, and even the extracellular vestibules, are indistinguishable. This suggests that domain swapping does not strongly affect the structural elements directly responsible for ion permeation and ion channel block, at least based on the comparison of the closed-pore states captured in the structures of TRPV6_cryst_ and TRPV6*. This conclusion was further supported experimentally by similar patterns of anomalous signals identified in the TRPV6_cryst_ and TRPV6* ion channel pores for calcium and gadolinium [43, 44].

### Conclusion

Although the first TRP channels were characterized over two decades ago [1, 2], the first structure of a nearly complete TRP channel was solved only recently, using advances in cryo-EM [21]. The delay in structure determination was a consequence of the difficulties in applying X-ray crystallography to TRP channels because of their inherent flexibility and multi-domain topology. Nevertheless, the advantages of X-ray crystallography over cryo-EM, especially the anomalous diffraction technique that can be used to visualize ions in a channel's pore, make it an indispensable tool for understanding the fundamental properties ion channels, including their permeation and block. Crystal structures of TRPV6 prove that X-ray crystallography can be used for TRP channel structure determination. Here we described methodology that we used in our attempts to crystallize TRPV6, especially focusing on construct engineering as the most efficient technique for obtaining diffraction quality crystals. Not only have we presented the experiments that produced positive results, but also those that yielded negative results. The latter, however, can produce positive results if applied to other membrane proteins and other representatives of the TRP channel family in particular. We hope that our experiments will inspire future crystallographic work on the most interesting and challenging targets. We believe that despite advances in cryo-EM, some answers to the vast number of questions regarding ion channel structure and function will still be provided by crystal structures obtained as a result of creative, persistent and fearless work.
